# Mechanotranduction Pathways in the Regulation of Mitochondrial Homeostasis in Cardiomyocytes

**DOI:** 10.3389/fcell.2020.625089

**Published:** 2021-01-21

**Authors:** Hongyu Liao, Yan Qi, Yida Ye, Peng Yue, Donghui Zhang, Yifei Li

**Affiliations:** ^1^Key Laboratory of Birth Defects and Related Diseases of Women and Children of Ministry of Education, Department of Pediatrics, West China Second University Hospital, Sichuan University, Chengdu, China; ^2^State Key Laboratory of Biocatalysis and Enzyme Engineering, School of Life Science, Hubei University, Wuhan, China

**Keywords:** mechanotransduction pathway, heart development, cardiac maturation, mitochondrial homeostasis, mitochondrial disorder

## Abstract

Mitochondria are one of the most important organelles in cardiomyocytes. Mitochondrial homeostasis is necessary for the maintenance of normal heart function. Mitochondria perform four major biological processes in cardiomyocytes: mitochondrial dynamics, metabolic regulation, Ca^2+^ handling, and redox generation. Additionally, the cardiovascular system is quite sensitive in responding to changes in mechanical stress from internal and external environments. Several mechanotransduction pathways are involved in regulating the physiological and pathophysiological status of cardiomyocytes. Typically, the extracellular matrix generates a stress-loading gradient, which can be sensed by sensors located in cellular membranes, including biophysical and biochemical sensors. In subsequent stages, stress stimulation would regulate the transcription of mitochondrial related genes through intracellular transduction pathways. Emerging evidence reveals that mechanotransduction pathways have greatly impacted the regulation of mitochondrial homeostasis. Excessive mechanical stress loading contributes to impairing mitochondrial function, leading to cardiac disorder. Therefore, the concept of restoring mitochondrial function by shutting down the excessive mechanotransduction pathways is a promising therapeutic strategy for cardiovascular diseases. Recently, viral and non-viral protocols have shown potentials in application of gene therapy. This review examines the biological process of mechanotransduction pathways in regulating mitochondrial function in response to mechanical stress during the development of cardiomyopathy and heart failure. We also summarize gene therapy delivery protocols to explore treatments based on mechanical stress–induced mitochondrial dysfunction, to provide new integrative insights into cardiovascular diseases.

## Introduction

The heart is an electromechanical organ that needs to beat thousands of times a day to provide enough blood supplement to the body (Saucerman et al., 2019). Cardiomyocytes (CMs) are subjected to chronic physiological hemodynamics, chamber pressure, tissue shape, and contractile stretch alterations (Barki-Harrington and Rockman, 2003; Linari et al., 2015; Lorenz et al., 2018). The Frank–Starling law and the Anrep effect describe the exquisite intrinsic mechanisms used by the heart to autoregulate contractile forces to maintain cardiac output under pre-load and afterload (Bluhm et al., 1998; Ait-Mou et al., 2016; Ruan et al., 2016). Throughout heart development, mechanical stress is essential for normal CM proliferation and differentiation during specification and morphogenesis (Miller et al., 2000; Clause et al., 2009; Banerjee et al., 2015). The heart follows a specific course of maturation, which begins from the very first heartbeat after birth, under hyperoxygenated conditions, and leads to the establishment of adult myocardial morphology (Guo and Pu, 2020). During heart development, there are dramatic adaptation switches involving gene expressions and the environment, including rapidly elevated circulating pressure. These changes require the maturation of CMs, which encode the physiological hypertrophy phenomenon (Gholipour and Tabrizi, 2020; Wang L. et al., 2020; Xiang et al., 2020). Following this, optimal levels of mechanical stress are involved in maintaining biological hemostasis and pathological maladaptation occurring in intracellular and extracellular matrix (ECM) remodeling (Kresh and Chopra, 2011; Collins et al., 2014; Dogan et al., 2016; Sessions et al., 2017).

In eukaryotic cells, mitochondria are involved in a large array of metabolic and bioenergetic processes that are vital for cell survival (Kaasik et al., 2004; Brown et al., 2010; Caffarra Malvezzi et al., 2020; Lyra-Leite et al., 2020). Within CMs, mitochondria are one of the most important organelles. Mitochondria are involved in almost all the major biological process of CMs, including supporting cellular morphology, ATP production through the electron transport chain (ETC), regulation of intracellular calcium ion signaling, and balance of reactive oxygen species (ROS) levels (Huss and Kelly, 2005; Koo and Guan, 2018; Fernandez-Caggiano et al., 2020). The mitochondria occupy a large fraction of CM cell volume (35–40%) and supply more than 90% energy of the cells' energy requirements. Over the last decade, we have seen an explosion in our knowledge of the role of mitochondrial dysfunction in human pathologies. This has led to the realization that mitochondria are important for all cell types and are especially important in energy-intensive cells including those of the skeletal and muscle, heart muscle, and neuronal cells. Emerging evidence indicates that mechanotransduction pathways greatly impact the regulation of mitochondrial homeostasis (Iribe et al., 2017). Excessive mechanical stress loading contributes to alterations in mitochondrial homeostasis, leading to cardiac dysfunction (Koo and Guan, 2018). The concept of restoring mitochondrial function by shutting down mechanotransduction pathways presents a potential therapeutic strategy for cardiovascular diseases. Recently, viral and non-viral protocols have shown great promise in regulating gene expression in CMs, highlighting the potential of these cells for gene therapy (Chen et al., 2016; Bezzerides et al., 2019; Wang S. et al., 2020). Herein, this review will highlight the biological processes of mechanotransduction pathways that respond to mechanical stress in CM dysfunction by regulating mitochondrial function.

## Biological Function of Mechanical Stress in CMs

Heart development involves (1) specification of cardiac progenitor cells, (2) formation of the linear heart tube, (3) cardiac looping, and (4) formation of the cardiac valve to form a mature beating heart. Normal mechanical stimulation is essential to maintain the normal physiological processes of CMs, including proliferation, differentiation, and maturation. Generally, there are three types of mechanical loading approaches to CMs, including shear stress, cyclic strain, and static stretching. Shear stress is generated by friction at the interface between the blood and the endocardium in the same direction as the blood flow (Lee J. M. et al., 2016). Cyclic strain is referred to the complex tensile and compressive strains with every heartbeat according to systolic and diastolic rhythm (Salameh et al., 2010). Static stretching should be considered as the compressive mechanical loading due to the blood pressure (Saucerman et al., 2019).

The physiological processes of CMs start at the beginning of mesoderm progenitor cell movements. These movements are initiated by a variety of morphogenic signals, including bone morphogenic protein (BMP), through the Wnt/activin/nodal pathway (Pandur, 2005; Murry and Keller, 2008; Ye et al., 2011), Gata-4 (Pu et al., 2004; Zeisberg et al., 2005; He et al., 2014; Akerberg et al., 2019), and the Hedgehog family (Mammoto and Ingber, 2010). Optimal stress force contributes to the maintenance of pluripotency through *Oct-4* expression (Fok and Zandstra, 2005; Earls et al., 2013). More recently, human induced pluripotent stem cells (iPSCs) have been successfully maintained at 6.4 dyn/cm^2^ for up to 32 days, with high levels of Oct4, Nanog, and alkaline phosphatase activity (Shafa et al., 2012). The two dominant types of stress during cardiogenesis are fluid shear stress and cyclic strain (Majkut et al., 2014). Even in the early stage of heart development during embryogenesis, influx and efflux of sodium and calcium trigger contractions while tube formation, indicating cyclic strain properties, is occurring (Sylva et al., 2014; Tyser et al., 2016). These are then followed by blood flow–induced pressure and shear stress. The functions of shear stress have been well-documented in endothelial cells. However, the role of shear stress during large parts of cardiomyogenesis remains unknown. In the embryonic stem cell model, *Mef2c* expression was induced under 10 dyn/cm^2^ shear stress (Kudo et al., 2000). Other studies indicate that the contributions of CMs, beyond endothelial cellular function, are very limited. Cyclic strain also maintains pluripotency in human embryonic stem cells and initiates the expression of key genes including *NOS-3, ET-1*, and *KLF-2* (Groenendijk et al., 2004). These genes regulate the differentiation of cardiac progenitors and illustrate the association between regions of increased differentiation and higher expression levels. Subjecting mouse embryonic CMs to cyclic stretch using an *in vitro* platform revealed that transforming growth factor β (TGF-β) plays a repressive role under these conditions (Banerjee et al., 2015). TGF-β is involved in the formation of hypoplasia of left heart syndrome via SMAD3 (Zeigler et al., 2016). Day 6 mouse embryoid bodies, exposed to 5–10% mechanical strain, have significantly increased levels of connexin 43 (Cx43) and Nkx2.5 expression (Schmelter et al., 2006; Gwak et al., 2008). Therefore, shear stress has important consequences in vascular network building and might be essential for the developing vasculature, whereas cyclic strain is critical for cardiomyogenesis.

Mechanical stimuli are involved in cellular physical structure formation from the membrane to nucleus. The ECM and cell–cell interaction allow intracellular connections to grow between the nuclear membrane and lamina (Jongsma and Wilders, 2000; Boukens et al., 2009; Delmar and McKenna, 2010). This contributes to isoform switching from lamin-B2 to lamin-A and controls the nucleocytoplasmic shunting of MKL1, a critical factor for cardiac functional maturation (Guo and Zheng, 2013, 2015; Ho et al., 2013; Guo et al., 2014). Cell–cell interaction between CMs involves the desmosome and adherens junction (Austin et al., 2019). This interaction includes *PKP2* (encoding plakophilin 2), *DSG2* (encoding desmoglein 2), *DSC2* (encoding desmocollin 2), *JUP* (junction plakoglobin), and *DSP* (desmoplakin) for desmosome formation and *CDH2* (encoding cadherin 2, also known as N-cadherin) and *CTNNA3* (encoding catenin-α3) for adherens junction formation (van Tintelen and Hauer, 2009; Sato et al., 2011; Saguner et al., 2013; Moncayo-Arlandi and Brugada, 2017; Austin et al., 2019; Kim et al., 2019; Xia et al., 2020). Importantly, mitochondria bind to microtubules and are affected by changes in their mechanotransmission. Ca^2+^ release by mitochondria is the first thing observed under mechanical stress. Iribe et al. demonstrated that stretch caused activation of the respiratory chain to hyperpolarize Δψm, followed by NADPH oxidase (NOX) activation, and increased ROS production (Iribe et al., 2017). Mechanical stress also controls mitochondrial fission and fusion, contributing to the post-natal maturation of mitochondria.

**Figure 1:**
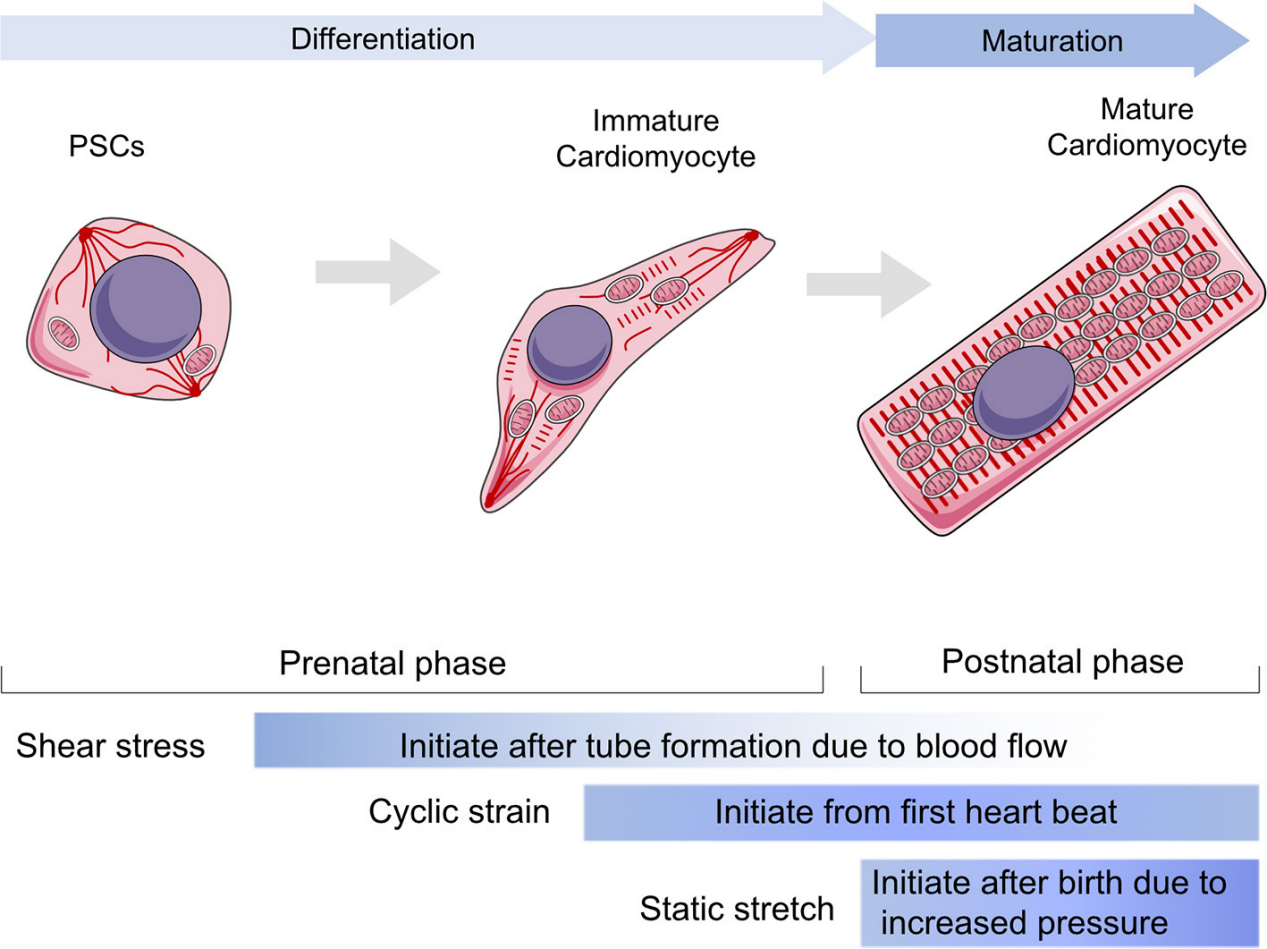
The role of mechanical stress in guiding cardiomyocytes in differentiation and maturation. The differentiation stage is referring the duration from progenitor stem cells to immature cardiomyocytes within prenatal phase, whereas the maturation stage is mainly referring to the post-natal phase to establish a functional cardiomyocyte. The shear stress would be loaded to cardiovascular system since tube formation, but it mainly impacts the early stage of cardiac development and vascular migrations. Cyclic strain influences cardiomyocytes after the heart starts to beat, while contributing to maintain heart function throughout all lifetime. The static stretch is considered to play a major role after birth as the pressure goes up rapidly and becomes the most dominant one following aging. PSCs, progenitor stem cells.

Mechanical stress is generated by circulating pressure and myocardium contraction, but is also related to ECM stiffness (Collins et al., 2014; Chiou et al., 2016; Herum et al., 2017). Moreover, cyclic strain helps to build a normal ECM around CMs by modulating fibroblasts (Saucerman et al., 2019). *In vivo*, paracrine signaling molecules are stimulated by mechanical stress and by targeting cardiac fibroblasts proliferation rates. The heart gradually grows larger and more functional as the body develops and matures (Guo and Pu, 2020). After the proliferation period, only a limited number of CMs have the ability to regenerate, and heart enlargement primarily occurs through CM hypertrophy. To maintain cardiac function and meet the mechanical demands of adapting to tissue stresses due to pressure or volume overload, CMs grow in size by elongating and making the myocardial wall thicker, which is a part of the maturation phase. In addition to shear stress and cyclic strain, static stretching becomes a dominant factor during the maturation phase (Aragona et al., 2013; Hirt et al., 2014; Karbassi et al., 2020). Hypertrophy is achieved by increasing cell volume and polarity and by the arrangement of contractile protein content and mitochondria. Additionally, mechanical loads affect the cytoskeleton or sarcomere to regulate cell shape and arrangement. Cardiac contractile force regulates the distribution of the vinculin (VCL) cytoskeletal protein and activates slingshot protein phosphatase 1 and the CFL actin depolymerizing factor to promote myofilament maturation via F-actin rearrangement (Guo and Pu, 2020). Recent studies showed that CMs from neonatal rats seeded on a collagen (Col)-coated PA gel matrix had an elastic modulus equal to 10 kPa, showing perfect morphological structure (Jacot et al., 2010). However, the sarcomeres of CMs seeded on matrix with an elastic modulus >10 kPa or smaller than 10 kPa were less defined and unaligned and contained stress fibers. Mechanical loads also regulate Ca^2+^ maturation in CMs. Ruan et al. used engineered myocardium under a static stretch of 0.63 ± 0.10 mN/mm^2^ and found increased expression of ryanodine receptor 2 and sarcoplasmic reticulum (SR)/endoplasmic reticulum (ER) calcium ATPase 2 (SERCA2) (Ruan et al., 2016). Cyclic mechanical stress during systole and passive stretch during diastole induce CM maturation in cell culture. In the process of myocardial contraction, the amount and duration of calcium ion release regulate the magnitude of contractile force (Zhang et al., 2020). Several studies have shown that the magnitude of the calcium transient and the amount of SR calcium and SERCA2a correlate with the magnitude of force generated in primary cultured neonatal rat CMs. Metabolic changes, including mitochondrial ETC function, morphology, and ROS production, are guided by mechanical stress. CM hypertrophy is also a key parameter of maturation post-natally (Caffarra Malvezzi et al., 2020). Inert polycaprolactone (PCL) planar layers with different cross-linking densities and Young modulus ranging from 1 to 133 MPa (measured by tensile test) have been prepared (Govoni et al., 2013). These PCL layers indicate that differentiation of the CM hypertrophic phenotype is influenced by substrate stiffness and that a more mature phenotype could be obtained with substrate stiffness of around 0.91 ± 0.08 MPa (Forte et al., 2012). The effect of substrate stiffness on the beating rate of CMs was also studied, and it was found that muscle cells beat fastest on substrates that mimic the stiffness of natural tissue. Additionally, the stretching substrate in mechanical stimulation can also promote the proliferation and maturation of functional CM characteristics. Therefore, CM development and maturation involve the physical cues of mechanical stress (Figure 1).

## Molecules Involved in Mechanotransduction in CMs

CMs are sensitive to mechanical stress, and responses to mechanical stress culminate in downstream gene transcription alterations. Therefore, the fundamental biological role of mechanotransduction is to transduce physical stimuli to molecular signaling. There are two common types of mechanical sensors within the cellular membranes of CMs, which are known as biophysical and biochemical sensor mediating pathways. The sensors are located in membrane to transduce extracellular stress stimulation into intracellular signals. Generally, the role of the physical sensors is to connect the ECM and cytoskeleton, to reshape actin proteins, and finally change chromosome structure to influence gene transcription. Chemical sensors mainly impact the modification of downstream molecules, to transduce signals into transcription regulation (Table 1).

**Table 1:** Molecules involved in mechanotransduction in CMs.

**Mechanotransduction**	**Function**	**Components**	**Signal pathways**
Biophysical sensors	Maintain cellular shape, Resist external mechanical force, Link up inner and outer cellular	Integrins, desmosome, adherens junctions	Akt, c-Jun N-terminal kinase, MAPK-ERK-p38
Biochemical sensors	Regular cardiogenesis and hypertrophic cardiomyopathy, maintain heart development under stress, Ca^2+^-channel regulation, mediating contraction–excitation coupling and heart rhythm	Ras-like small GTP-binding proteins, Ras superfamily including Ras, Rho, Ran, and Rab families	RAS/RAF-dependent MAPK signaling, RhoA signaling RAP-Hippo-YAP pathway

### Biophysical Sensors

**Figure 2:**
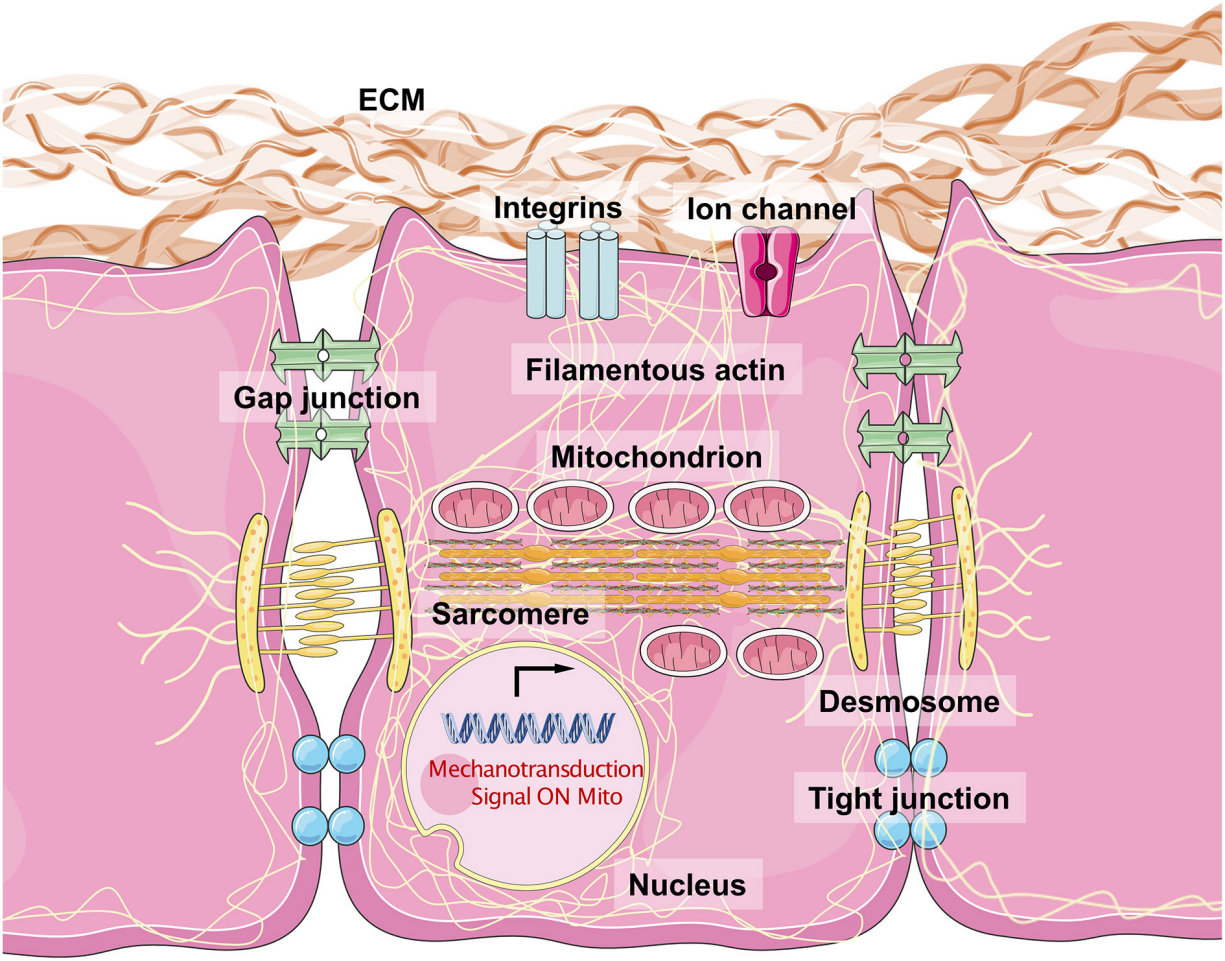
The biophysical sensors of mechanical stress in cardiomyocytes and the intracellular mechanisms. The integrins are considered as the most major biophysical sensors located in cellular membrane of cardiomyocytes. While the desmosome, gap junction, and tight junction also contribute to maintain a cell–cell junction with a physical mechanical contact. Filamentous actin connects all the molecules with mitochondria, sarcomere, and nuclear membrane, especially lamin A/C. The acto–myosin action and myosin II structure make the dominant unit for actin movement. Once the mechanical stress loaded on cell surface, the integrin would phosphorylate FAK and downstream molecules, which force the myosin protein to move along acto–myosin actin, and then change the shapes of mitochondria, sarcomere, and nuclear membrane, regulating related genes expression and mitochondrial function.

The sensing of mechanical force and the transduction of the resulting signal are a typical physical process. It is important that CMs resist external mechanical force to maintain their normal cellular shape and initiate crosstalk between inner-cellular and outer-cellular forces (Geiger et al., 2009; Oria et al., 2017). The ECM surrounds the CMs to provide an optimal microenvironment for myocardiogenesis and maturation. Typically, the major structural components of cardiac ECMs include fibrillar Col, fibronectin (FN), glycoproteins, proteoglycans, and glycosaminoglycans (Rienks et al., 2014). Fibroblasts critically contribute to the generation of ECM macromolecules. The architectural meshwork of the ECM also works as a reservoir for cytokines including TGF-β, BMP, platelet-derived growth factor, and connective tissue growth factor (Carè et al., 2007; Gordon and Blobe, 2008; Francisco et al., 2020). The proportion of different Cols (*Col1a1a, Col1a2*, and *Col5a1*) and cytokine expression levels contribute to the changes in ECM stiffness observed in healthy and damaged myocardium tissues (Horn and Trafford, 2016). Integrins are the major substrates that connect the ECM to the costamere proteins, establishing a bridge from the extracellular environment to the Z-line located in the sarcomere (Wang et al., 1993). Several integrins, consisting of α and β subunits, are expressed in mammalian cells. α1, α5, α7, and β1 are the most highly expressed subunits in CMs and form α1β1, α5β1, and α7β1 heterodimers, which are predominantly Col, FN, and laminin-binding receptors (Israeli-Rosenberg et al., 2014). This kind of structure mediates the connection between CMs and the ECM, while the desmosome (including PKP2, DSG2, DSC2, JUP, and DSP) and adherens junctions (including CHD and CTNNA3, which mainly serve as types of cadherins) maintain cell–cell adhesions, which facilitate the binding of desmin to intermediate filaments. Structural proteins on the cell surface bind to filamentous actin (acto–myosin stress fiber and α-actin) and myosin (myosin II), generating intracellular force to target the nuclear membrane (Maniotis et al., 1997; Geiger and Bershadsky, 2002). Thus, cytoskeletal force generation helps to push against the stress from the ECM, inducing a bidirectional balance that maintains normal cellular shape. Increasing force stretches the structural proteins, and once mechanical force exceeds the threshold, activation domains are exposed, recruiting talin to the β subunits of integrins, VCL, paxillin, and FA kinase (FAK). This produces a wide range of intracellular signals, including those of the Akt, c-Jun N-terminal kinase, and MAPK-ERK-p38 pathways (Sun et al., 2019). This biophysical mechanotransduction pathway has been presented simply in Figure 2.

### Biochemical Sensors

Biochemical sensors are biomechanical stress–sensitive activators that regulate CMs. Ras-like small GTP-binding proteins are the main components of myocardial biochemical sensors. The Ras superfamily, including Ras, Rho, Ran, and Rab families, is involved in cardiovascular diseases. The transition between the GTP- and GDP-bound forms of Ras proteins is accompanied by conformational changes that significantly affect its affinity for downstream signaling molecules (Meng et al., 2018). The activation of the RAS/RAF/MEK/ERK1/2 cascade is essential for cardiogenesis, and RAS/RAF-dependent MAPK signaling induces hypertrophic cardiomyopathy. RhoA signaling activates SRF and Mef2c to maintain heart development under stress. Moreover, a large number of Ras superfamily proteins are involved in Ca^2+^-channel regulation, mediating contraction–excitation coupling and heart rhythm. Kluge et al. demonstrated that the Rho-family GTPase1, Rnd1, serves as a biomechanical stress–sensitive activator, influencing cell proliferation, and cellular hypertrophy via activation of RhoA-mediated SRF-dependent and -independent signaling pathways (Kluge et al., 2019).

**Figure 3:**
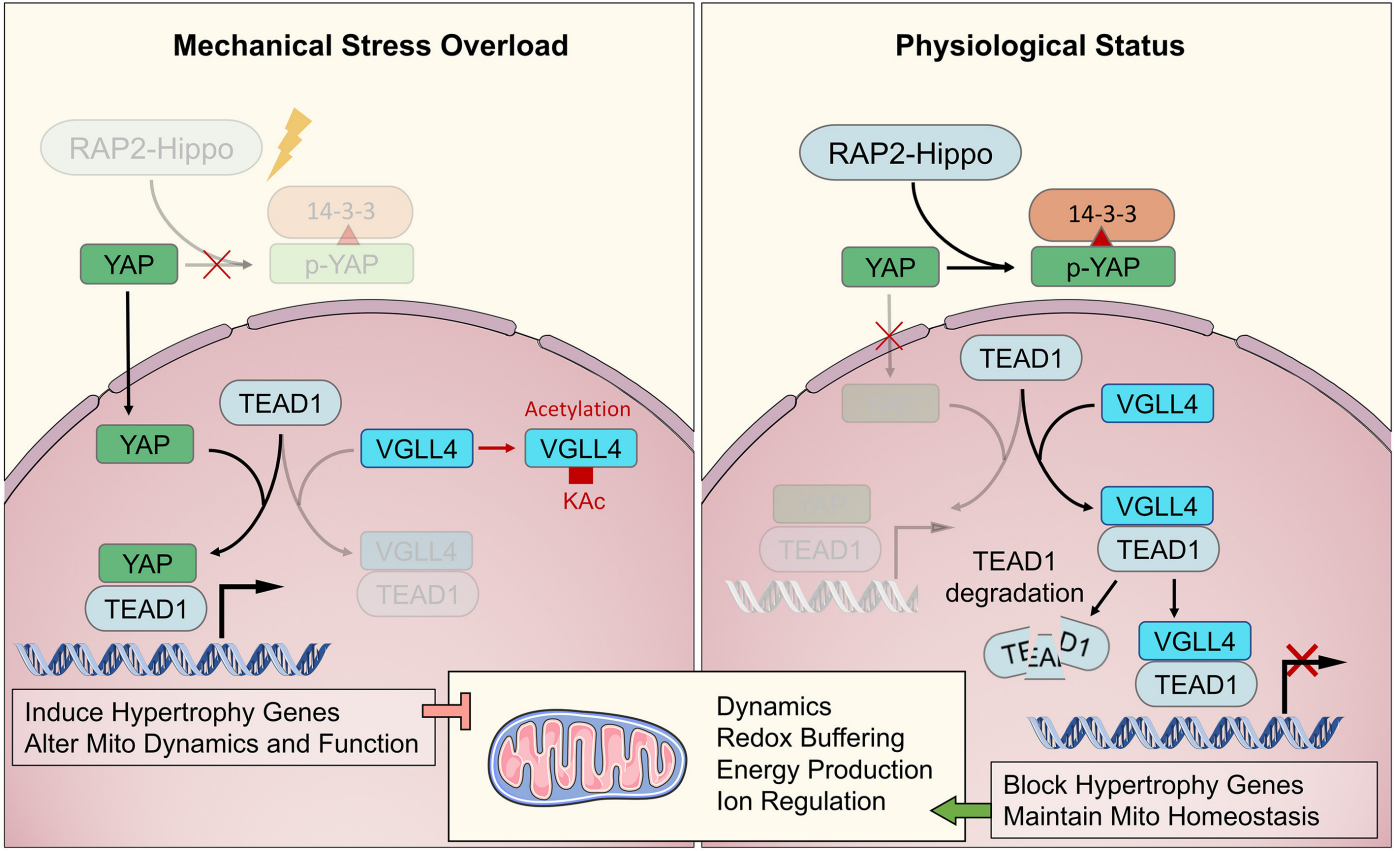
The major biochemical pathway of RAP2-Hippo-YAP in mechanical transduction. The RAP2-Hippo-YAP pathway is the most determined one of biochemical sensor pathway. The overloading pressure would inactivate the RAP2 and then turn off Hippo signaling. Then, the YAP would be dephosphorylated and translocated in to nucleus. VGLL4 demonstrated a competition role with YAP in binding to TEAD1. The exceeding mechanical stress would activate YAP and up-regulated hypertrophic genes, leading to mitochondrial dysfunction in adulthood.

Recently, Meng et al. (2018) demonstrated that Rap2 is a key intracellular signal transducer that controls mechanosensitive cellular activities through Yes-associated protein (YAP) and transcriptional coactivator with PDZ-binding motif (TAZ). Mechanistically, matrix stiffness influences the levels of phosphatidylinositol 4,5-bisphosphate and phosphatidic acid through phospholipase Cγ1 (PLCγ1), leading to Rap2 activation through PDZGEF1 and PDZGEF2. Therefore, Rap2 is a pioneer that converts mechanical signals from physical stress to biochemical molecular activation. Deletion of *RAP2* increases the nuclear localization of YAP and TAZ. However, the functions of Rap2 have not been confirmed in cardiogenesis and maturation, or in cardiomyopathy. The Hippo-YAP/TAZ pathway functions downstream of Rap2 and has been well-studied throughout heart biogenesis, hemostasis, and regeneration. Under normal mechanical stress, activated Hippo pathway proteins, including MAP4K4, MAP4K7, ARHGAP29, LATS1/2, and MOB1/2, phosphorylate YAP/TAZ, confining them to the cytoplasm (Dupont et al., 2011; Aragona et al., 2013). Under excessive mechanical stress, Hippo is deactivated, and YAP/TAZ translocate into the nucleus as co–transcription factors that interact with TEAD1 family proteins to develop transcription complexes (Mosqueira et al., 2014; Totaro et al., 2018). TEAD1 functions in cardiogenesis during the fetal stage of development and regulates the actin cytoskeleton and metabolism in CMs during the adult stage. VGLL4 binds with TEAD1 to regulate YAP function to control downstream genes. When VGLL4 binds to TEAD1, it induces TEAD1 degradation, inhibiting the response to exceeding mechanical stress (Kim et al., 2015; Narimatsu et al., 2015; Chang et al., 2018). The RAP-Hippo-YAP pathway is illustrated in Figure 3.

Moreover, the biophysical and biochemical sensors demonstrated several instances of crosstalk in their downstream signals. The biophysical transduction pathways have been studied for several years, and functional molecules have been identified. However, we still have very limited knowledge about biochemical sensors and their functional messengers. The key molecules, YAP/TAZ, are involved in both biophysical and chemical regulation pathways. Therefore, we believe there should be more connection between the two sensor types. However, there is still no evidence that shows how one kind of sensor induced pathway changes when another is knocked out. ECM remodeling is important for the maintenance of CM function. Therefore, it is necessary to understand how biochemical sensors influence the ECM, which is always bound by integrins, to regulating biophysical signaling.

## The Common Understanding of Mitochondrial Homeostasis in CMs

### Mitochondrial Quality Control

Mitochondria maintain their shape and number through biogenesis, fission, fusion, and mitophagy to ensure normal physiological function. This process is called mitochondrial quality control (MQC). MQC is a complex process that includes three main levels. The first level prevents mitochondrial damage and maintains mitochondrial stability through proteasome activation. The second level maintains the number and shape of mitochondria through mitochondrial biogenesis, fission, and fusion. The third level selectively removes damaged mitochondria and is called mitophagy (Pickles et al., 2018).

#### Biogenesis and Degradation

Mitochondrial biogenesis is the process through which cells increase their mitochondrial mass. Proliferator-activated receptor γ coactivator 1 (PGC1) is a critical factor in this process. PGC1 activates peroxisome proliferator-activated receptor, which leads to the gain of mitochondrial DNA (mtDNA) content, and promotes the transcription of mitochondrial uncoupling protein 1 (UCP1). This helps CMs adapt to the post-natal environment with a mature ETC utilizing fatty acid. PGC-1α expression is regulated by YAP/TAZ, and PGC-1α depletion results in lethal cardiomyopathy. Moreover, PCG-1 interacts with Mfn1/Mfn2 and Drp1. TFAM is another essential factor for mitochondrial biogenesis and is required for mtDNA transcription and mitochondrial self-replication. Zhang and Li demonstrated that the Nkx2.5-driven Tfam knockout leads to total mitochondrial loss in CMs (Zhang et al., 2018).

Mitochondria are divided into four parts by the double-layer membrane structure, the mitochondrial outer membrane, mitochondrial inner membrane, intermembrane space, and matrix. In the mitochondrial outer membrane, the ubiquitin–protease degradation system (UPS) is the main mechanism of maintaining homeostasis. The UPS consists of ubiquitin, ubiquitin initiation enzyme, the 26S proteasome, and deubiquitination enzyme. Ubiquitin initiation enzyme is responsible for activating ubiquitin and target protein binding to form a target protein polyubiquitin chain or to ubiquitinate the degraded protein. Then, ubiquitinated proteins are transported to 26S proteasomes, where they are recognized and degraded (Tsakiri and Trougakos, 2015). The deubiquitination enzyme dissociates ubiquitin from the substrate, allowing ubiquitin recycling (Fang et al., 2010). UPS is involved in abnormal protein degradation and plays an important regulatory role in the cell cycle, signal transduction, DNA damage repair, and cell stress. Impairment of UPS function has been detected in heart tissues of patients with hypertrophic cardiomyopathy and heart failure (Predmore et al., 2010). Conversely, enhancement of cardiac proteasome proteolytic function plays a protective role against the pathophysiology of proteinopathy and ischemia–reperfusion (IR) injury in mice (Li et al., 2011). Furthermore, accumulation of polyubiquitinated substrates has been observed in the heart tissue of patients with cardiac diseases such as cardiomyopathy and heart failure (Nishida and Otsu, 2017).

In the intermembrane space, HTRA2/OMI is thought to function as a protein quality control protease. HTRA2/OMI is an ATP-independent serine protease involved in the regulation of mitochondrial E3 ubiquitin ligase. Lack of HTRA2/OMI leads to mitochondrial dysfunction, mitochondrial morphological changes, and the production of ROS, leading to mtDNA damage. There are two ATPases associated with various cellular activities (AAAs) in the mitochondrial inner membrane. The proteasome complex can recognize misfolded transmembrane protein polypeptide chains and degrade unassembled mitochondrial complex subunits and transmembrane segments (Leonhard et al., 2000). The biochemical and genetic interactions of the RUVBL1 and RUVBL2 AAA family proteins play a novel functional role in symmetry breaking and cardiac development (Hartill et al., 2018). There are two AAA proteins in the mitochondrial matrix, Lon and ClpXP. Lon is mainly responsible for removing oxidized proteins and preventing oxidative stress damage. ClpXP can degrade proteins that are not bound to molecular chaperones and mediate the mitochondrial unfolded protein reaction (Hammerling and Gustafsson, 2014). Lon is involved in cardiac disorders. Lon is upregulated in hypoxia-induced CMs, and Lon downregulation attenuates hypoxia-induced CM apoptosis through decreased oxidant generation. Lon overexpression stimulates oxidant production and induces apoptosis under normoxic conditions in CMs (Bota and Davies, 2016).

The selective removal of redundant or damaged mitochondria through the process of autophagy is called mitochondrial autophagy or mitophagy. The selective removal of damaged mitochondria is a complex process whose molecular mechanisms involve a variety of proteins, which are mainly classified as Parkin-dependent or Parkin-independent. Parkin-dependent mitophagy is the most dominant mechanism of mitophagy. The transfer of Parkin requires PTEN-induced kinase 1 (PINK1). PINK1 recruits phosphorylated Parkin through its silk/threonine kinase activity and transfers Parkin from the cytoplasm to the mitochondria. Parkin has E3 ubiquitin ligase activity and can mediate the ubiquitination of mitochondrial outer membrane proteins including HKI, VDAC1, and MFN1/2, to initiate mitophagy (Geisler et al., 2010). Parkin-independent regulatory proteins include the BNIP3, NIX, and FUNDC1 mitophagy receptors. FUNDC1 is highly expressed in the heart, interacts with LC3 on autophagosome membranes, and mediates mitochondrial fragmentation and mitophagy under hypoxia (Liu et al., 2012). BNIP3 and NIX are proapoptotic proteins located in the outer membrane of mitochondria and can directly bind with LC3 to induce mitochondrial recruitment into autophagosomes for degradation. In a Parkin knockout fly model, CM mitochondria exhibit dysmorphology, depolarization, and ROS generation. Accumulation of enlarged hollow donut mitochondria with dilated cardiomyopathy was also observed and could be rescued by CM-specific Parkin expression (Bhandari et al., 2014). Furthermore, Parkin deletion led to increased DRP1 levels and simultaneous deficiency of DRP1 and Parkin-exacerbated cardiomyopathy (Kageyama et al., 2014). In 2017, it was reported that resveratrol, known for its antiaging properties, can be used to treat cardiovascular complications related to aging through Parkin and PINK1 activation (Ren et al., 2017). However, mitophagy can be also detrimental. Some findings demonstrate that when CMs undergo IR, ATP production is reduced because of greatly enhanced mitophagy and impaired mitochondrial fission and fusion leading to cell damage (Anzell et al., 2018).

#### Fission and Fusion

Mitochondrial fission and fusion must exist in balance to maintain mitochondrial integrity, which is critical for heart health. Through mitochondrial fission, the required number of mitochondria can be ensured, irreversible mitochondrial damage can be isolated, and mitochondrial movement and distribution can be promoted. Mitochondrial fission is mainly mediated by dynamin-related protein 1 (DRP1). DRP1 is highly expressed in heart tissues and can be upregulated under stress. Additionally, post-transcriptional SUMOylation and phosphorylation modulate DRP1 function. Mitochondrial fusion involves the joining of normal and damaged mitochondria to replace the materials in damaged mitochondria. This is conducive to material circulation and energy transmission and can protect cells. Mitochondrial fusion is mainly mediated by mitochondrial fusion proteins, including MFN1 and MFN2, which serve the outer mitochondrial membrane, and optic atrophy 1 (OPA1), which serves the intermitochondrial membrane. MFN1 and MFN2 have amino-terminal conserved GTPase domains and carboxy-terminal coiled helix structures and can bind to the mitochondrial outer membrane and form transhomologous or heterologous oligomeric complexes simultaneously, bringing two mitochondria into close proximity for fusion (Koshiba et al., 2004). Proteins involved in fission and fusion function within a signaling network to maintain mitochondrial homeostasis and play important roles in regulating cardiac response under pathological stress and mechanical stretching, such as during IR, cardiomyopathy, and heart failure (Kuzmicic et al., 2011). For example, DRP1 ablation in adult mouse cardiac myocytes interrupts mitochondrial fission and provokes the mitophagic mitochondrial depletion that contributes to lethal cardiomyopathy (Song et al., 2015a). Deletion of Mfn1/Mfn2 fusion proteins in mouse heart results in abnormal mitochondrial morphology and mitochondrial fragmentation, leading to ventricular wall thickening and an increase in cardiac mass (>30%) accompanied by symptoms of eccentric hypertrophy. Additionally, the Mfn1/Mfn2/Drp1 triple knockout causes mitochondrial heterogeneity and impaired mitophagy (Song et al., 2015b). Moreover, OPA1 helps to maintain the cristae structure and reduce apoptosis.

### ATP Production

Mitochondria use fatty acids, glucose, and amino acid metabolites to synthesize ATP through the tricarboxylic acid cycle and oxidative phosphorylation pathways. ATP production through the ETC is the principal function of mitochondria. The ETC is a multisubunit complex in the mitochondrial inner membrane and is an important field of ATP production. The transportation of protons from the matrix to the intermembrane space through complexes I, III, and IV generates energy. The mitochondrial transmembrane potential (Δψm) is essential to maintain the electrochemical proton gradient across the mitochondrial inner membrane and should always remain 150–180 mV negative to the cytosol. ATP produced by mitochondria in myocardial tissue is mainly responsible for myocardial contraction, cellular excitability, and maintenance of calcium homeostasis. McCully et al. found that under myocardial ischemia and other pathological conditions, mitochondrial respiratory chain function is decreased, and ATP synthesis is impaired (McCully et al., 2007). Moreover, in some genetic mitochondrial diseases, the CMs reveal increased basic energy production, but deficient maximal ATP synthesis (Zhang et al., 2018). In turn, ATP synthesis disorder leads to changes in various biochemical and ultrastructure properties of CMs, including calcium accumulation and acidosis in the cytoplasm, mitochondria, and nucleus, as well as large quantities of ROS production, leading to progressive damage of mitochondrial function and decreased CM activity.

### Regulation of ROS

Normally, the respiratory chain effectively uses more than 98% of the electrons to synthesize ATP, and 1–2% of the electrons are released outside the mitochondria to produce ROS. ROS are involved in signal transduction and regulation of apoptosis in cell response to stress and risk factors and are broken down by superoxide dismutase (Penna et al., 2009). In pathological conditions, the uncoupling of oxidation and phosphorylation results in a mass release of electrons. Excessive ROS production then leads to oxidation of mtDNA, lipids, and proteins and extensive cell damage. MtDNA is in a state of continuous replication with weak DNA repair abilities, making it vulnerable to oxidative damage. MtDNA damage further stimulates the production of ROS, forming a vicious cycle and aggravating the damage of CMs (Yu and Bennett, 2014; Lee et al., 2017). ROS regulation also plays an important role in heart development, whereas mitochondrial damage would cause ROS accumulation, which induces DNA damage and leads to cell cycle arrest (Zhang et al., 2018).

### Ca^2+^ Handling

Mitochondrial Ca^2+^ homeostasis is a result of the dynamic equilibrium between Ca^2+^ influx and efflux. Many factors influence these processes. First, the spatial organization of mitochondria within cells and their tethering with other organelles, especially with the ER, play a pivotal role in mitochondrial Ca^2+^ homeostasis. The estimated area of contact sites between mitochondria and ER accounts for 5–20% of the total mitochondrial surface (Rizzuto et al., 1998). It is broadly considered that these contact sites are very important and are the sites where Ca^2+^ is rapidly transported into the matrix. De Brito and Scorrano identified MFN2 as an essential protein that directly bridges these two organelles, and mitochondria Ca^2+^ uptake is impaired following genetic ablation of MFN2 (de Brito and Scorrano, 2008). Second, the uptake of calcium by mitochondria depends on the MCU (mitochondrial Ca^2+^ uniporter). The electrophysiological characterization and molecular identity of MCU are partially determined. MCU is an inward rectifying channel that is highly Ca^2+^-selective (Kirichok et al., 2004). Mitochondrial protein MICU1 (mitochondrial Ca^2+^ uptake protein 1), SLC25A23 (solute carrier 25A23), and MCUR1 (mitochondrial calcium uniporter regulator 1) are important regulators of Ca^2+^ uptake (Perocchi et al., 2010; Sancak et al., 2013; Hoffman et al., 2014; Mallilankaraman et al., 2015). A growing number of researchers found that MCU is a key component of a higher-order macromolecular complex that requires further investigation but was named the MCU complex (De Stefani et al., 2015). Furthermore, Ca^2+^ release from mitochondria relies on two different pathways, the 2H^+^/Ca^2+^ antiporter (mHCX), and the Na^+^/Ca^2+^ exchanger (NCLX) (Palty et al., 2010).

## Mechanical Cues Contribute to the Maintenance of Mitochondrial Homeostasis

### Mechanical Stress Controls MQC

In normal conditions, the mechanisms of MQC mainly consist of proteasome activation, fission and fusion, and mitophagy. Furthermore, mitochondria exhibit some unique behaviors when cells undergo mechanical stress. In the heart, abnormal mechanical stress is mainly caused by hypertension, ventricular hypertrophy, ECM remodeling, or other types of cardiomyopathy. As rich contents and ATP factories of CMs, mitochondria will show corresponding pathophysiological changes. A study in rats with spontaneous hypertension and left ventricle hypertrophy reported overexpression of proteins related to mitochondrial oxidative phosphorylation and underexpression of the mitochondrial precursor of ATP synthase (Zamorano-León et al., 2010).

The mechanisms of MCQ are also important. Alterations in mitochondrial fission, fusion, and mitophagy are associated with pathological conditions in the heart. DRP1 is an essential protein that mediates mitochondrial fission under stress. Hypertension is usually accompanied by elevated levels of norepinephrine. Using norepinephrine to culture CMs of newborn rats can promote mitochondrial fission. This is because norepinephrine can increase cytoplasmic Ca^2+^ and activate calcineurin to promote DRP1 migration to mitochondria (Pennanen et al., 2014). After DRP1 has been recruited to mitochondria, the phosphorylation of the GTPase effector domain of DRP1 at Ser637 reduces its response to norepinephrine, which causes a further increase in mitochondrial fission (Santel and Frank, 2008). Taken together, these data indicate that mitochondrial fission may be a compensatory mechanism to maintain heart contractility under conditions of exceeding mechanical stress (Lahera et al., 2017). However, mitochondrial fusion is a repressed process under stress. A study demonstrated a decrease in mRNA levels of Mfn1 and Mfn2, which are major proteins mediating fusion in hypertensive and cardiac hypertrophy rats caused by phenylephrine (Fang et al., 2007). Mitophagy is another essential mechanism to maintain MCQ. However, under pressure overload, mitophagy can be maladaptive. Sympathetic, parasympathetic, renin–angiotensin–aldosterone, and antidiuretic hormone systems are involved in blood pressure regulation. And most peptides and hormones in these systems can regulate mitophagy (Gottlieb and Thomas, 2017). For example, the angiotensin receptor blocker valsartan can diminish mitophagy (Zhang et al., 2014). Usually, mitophagy is enhanced in the heart of patients with hypertension, and hemodynamic stress can induce a robust autophagic response in cardiac myocytes (Zhu et al., 2007), which is considered detrimental. Mechanical stress can upregulate *Beclin-1* gene expression, which amplifies the autophagic response to stress and augments pathological remodeling (Zhu et al., 2007). However, the absence of mitophagy is also a type of maladaptation. Temporally controlling cardiac-specific Atg5 deficiency in mice suggested that autophagy plays a beneficial role in the response of the heart to pressure overload and is important for preventing the accumulation of abnormal proteins or damaged organelles (Nakai et al., 2007). Mechanical stress regulates mitophagy to maintain optimal mitochondrial number and health.

### Mechanical Transduction Pathways in the ETC

Normal physiological processes of the cell are accompanied by ATP consumption. CMs need a large amount of ATP due to their contraction activity. To make more efficient use of ATP to support CMs, many mitochondria attach to the sarcomere and SR (Pasqualini et al., 2016). This arrangement greatly promotes the transport of intermediate metabolites in nucleotide and oxidative phosphorylation (Auerbach et al., 1997).

In the early stage of mechanical stress, CM structure is intact, energy metabolism is mainly performed by fatty acid as substrate, and sufficient ATP is produced through the ETC to supply normal mechanical CM contraction (Lopaschuk et al., 2010). However, after a longer period of mechanical stress, the mitochondrial structure varies with abnormal MQC, the ETC is impaired, glycolysis and glucose oxidation increase, and the energy supply becomes inadequate. The utilization of energy substrates and changes in energy metabolism, including abnormal mitochondrial function and increased glucose utilization, are phenotypes of myocardial hypertrophy and heart failure (Ventura-Clapier et al., 2004; Stanley et al., 2005).

External mechanical stress activates the integrin signaling pathway mediated by MAPK (de Cavanagh et al., 2009) and Rho-family GTP-binding proteins, and the stress response damages the mitochondrial ETC (Werner and Werb, 2002). Following mechanical stress, the heart gradually loses the ability to produce an adequate ATP supply, leading to heart failure. Mechanical force and cell junctions can regulate RhoA and affect YAP and TAZ by LATS-dependent or independent mechanisms (Zhao et al., 2012). YAP and TAZ can regulate UCP1, Marf, Opa1, and many glutamine-metabolizing enzymes, which are coupled with ATP production (Kashihara and Sadoshima, 2019).

### Exceeding Mechanical Loading Induces ROS Accumulation

Exceeding mechanical loading can occur via several methods, including cyclic strain, shear stress, and static stretch. CMs seeded on BioFlex culture plates were placed on a gasketed baseplate and subjected to a vacuum of −5 or −21 kPa using a Flexcell system, low and high tension, and a frequency of 1 Hz to generate cyclic strain. Using these parameters, the system generates a deformation gradient on the membrane, with maximum deformation of 5 and 25% at −5 and −21 kPa, respectively. In this system, short-term mechanical stress results in increased CM contractile force *in vitro*, whereas long-term and transient stimulation leads to structural changes in CMs (Pedrozo et al., 2015). Different stretching amplitudes in neonatal rat ventricular cells cause ERK1/2 phosphorylation, but only high-intensity stretching causes CM apoptosis and JNK phosphorylation. Activation of ERK1/2 and JNK is also accompanied by increased ROS. Single-cell stretch, known as the carbon fiber method, is another simple stretch method for cyclic strain. In brief, a pair of carbon fibers (10 μm in diameter) are attached to either end of a CM using custom-made three-axis hydraulic manipulators, each mounted on separate computer-controlled piezo-electric translators on a custom-made railing system (Pimentel et al., 2001). Another study found that excessive mechanical stress activates RAC1-ROS and leads to ROS accumulation in rat ventricular cells. This accumulation induces apoptosis and activates the downstream p38-MAPK pathway, eventually leading to pathological hypertrophy of the cell (Aikawa et al., 2001). Experimental analysis showed that mechanical stretching can lead to increased ROS production, cytoskeleton changes, apoptosis, and necrosis (Ulmer and Eschenhagen, 2020). Similar conclusions were reached in studies using rat and human CMs (Fujita and Ishikawa, 2011; Mohamed et al., 2016; Iribe et al., 2017).

### Regulation of Ca^2+^ Transit Under Mechanical Stress

Ca^2+^ affects CM contraction and is affected by mechanical stress (Bers, 2008). Ca^2+^ transit is difficult to observe and analyze *in vivo*. Therefore, mechanical stress models established *in vitro* allow for the visualization of Ca^2+^ transit. Cells isolated from human heart failure hearts show a decrease in the amplitude of Ca^2+^ transients with reduced Ca^2+^ removal rates (Lou et al., 2012). *In vitro* mechanical stress largely consists of large amplitude and large force or small amplitude and small force (Kurihara and Komukai, 1995). Axial stretching reduced the total Ca^2+^ load of guinea pig CMs within seconds (Iribe and Kohl, 2008). Another study showed that stretching increased the Ca^2+^ sparks rate of rat CMs through a nitric oxide–mediated pathway after prolonged exposure to mechanical stimulus (Petroff et al., 2001). A study in neonatal mouse CMs showed that the Ca^2+^ signaling pathway is mainly involved in the early stages of stretch-induced apoptosis. Ca^2+^ signaling pathways are located upstream of these known stretch-activated apoptotic events, such as caspase 3/9 activation, mitochondrial membrane potential corruption, and ROS production. Inhibition of intracellular Ca^2+^ can prevent these events (Liao et al., 2003). Additionally, researchers found that Duchenne muscular dystrophy (mdx) is a model of mechanical stress that triggers Ca^2+^ responses in resting dystrophy mdx CMs. Following mechanical stretch, multiple Ca^2+^ influx pathways are activated by stretch-activated channels, resulting in abnormal Ca^2+^ responses in mdx myocytes (Fanchaouy et al., 2009). In a study examining the acute effect of the axial application of single rat CM on the diastolic Ca^2+^ discharge rate, it was found that a rapid but short-term calcium spark was guided through a pathway that required the integrity of the cytoskeleton (Iribe et al., 2009). Within a few minutes of the pressure increasing, Ca^2+^ participated in the accumulation of the Anrep effect through autocrine/paracrine signals induced by stretching (Cingolani et al., 2013). When the stress is chronic, the increased calcium influx activates CaMKII/MEF2 expression and leads to cardiac hypertrophy (Gómez et al., 2013). Prosser et al. (Prosser et al., 2011) demonstrated that heart cell stretching activates NOX2 and leads to ROS generation in a microtubule-dependent manner under healthy conditions. This is known as “X-ROS signaling” and describes a mechanochemical signal induction of ROS-mediated RYRs and SR activation to generate a calcium burst, leading to muscle contraction (Santulli et al., 2018). In diseased CMs of Duchenne muscular dystrophy, “X-ROS signaling” generates arrhythmogenic Ca^2+^ waves (Khairallah et al., 2012). Exceeding mechanical stress conditions imply high concentrations of peroxynitrite, which causes SERCA2a inactivation through nitration. Lokuta et al. revealed that SERCA2a inactivation by nitration might contribute to calcium pump failure (Lokuta et al., 2005).

## Targeting Mechanotransduction Pathways to Restore Mitochondrial Function

**Figure 4:**
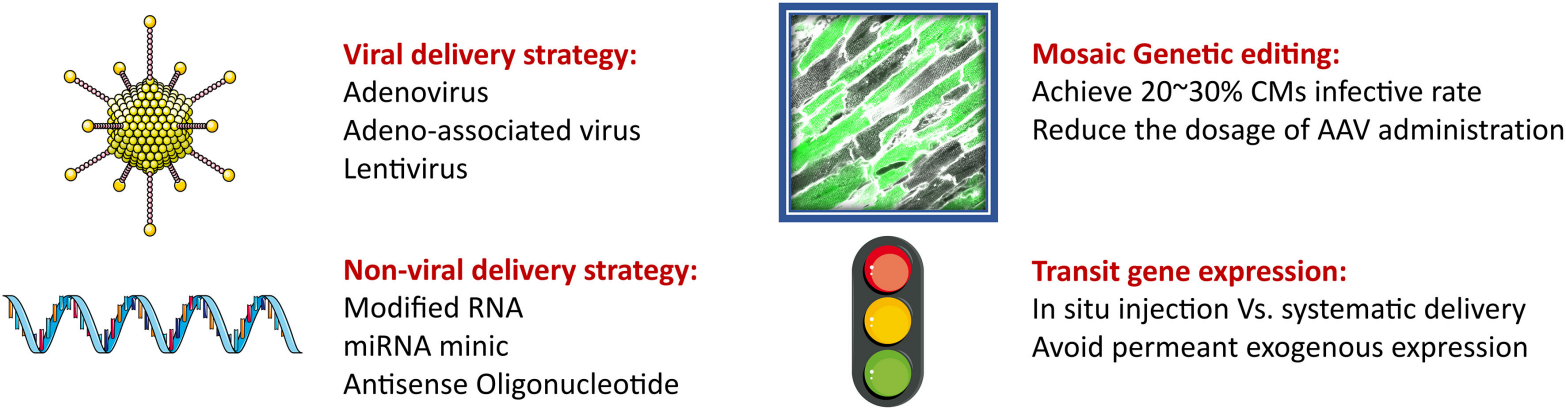
The strategy for gene delivery for CMs therapy. Gene therapy is a promising way to transduce exogenous gene expression. The two most common delivery methods are viral and non-viral vehicles. AAV is the most promising vector to delivery oligonucleotides. However, risk of high dosage of AAV administration limits its clinical practice. Thus, the mosaic genetic editing has been brought to reduce the requirement for AAV to avoid adverse effects. Besides, the non-viral delivery strategy is able to do transit gene expression, which works as an alternation to viral approach. Intramyocardial *in situ* and systematic injections are both able to achieve therapeutic goal.

Exceeding mechanical stress induces the dysfunction of myocardial mitochondria, leading to morphological changes and contractile impairment. Therefore, blocking abnormal mechanotransduction appears to be a valid approach of attenuating such adverse effects. However, this strategy brings a new issue into consideration: How can we block mechanotransduction when normal mechanical stress helps to maintain CM hemostasis? Therapeutic strategies targeting the mechanotransduction pathway should avoid the development and maturation stage, where a lack of mechanical stress would induce myocardium hyperplasia. Besides, the total knockout of any key genes in transduction pathway seems unacceptable. However, it has recently been demonstrated that the deletion of a specific gene in around 30% CMs would achieve the therapeutic goal. Following this, a method called “mosaic” knockout was developed to generate partial knockout CMs using varied dosage of delivery vehicles, including adeno-associated virus (AAV) (Guo et al., 2017; VanDusen et al., 2017). In the past year, the risks associated with AAV usage have been raised. Therefore, instead of generating permanent isogeneic insertions, some non-viral transient gene expression technologies have been developed that alter expression using modified mRNAs, miRNAs, and non-coding RNAs (ncRNAs) (Figure 4).

### Small Molecular Inhibitors

Recently, many reports have focused on the development of therapeutic compounds and drugs targeting mechanotransduction pathways to restore mitochondrial function in heart disease. For example, apigenin inhibits ROS generation, the loss of mitochondrial membrane potential, and apoptosis through PI3K/AKT signals and mitochondrial Notch1/HES1 signals, protecting H9C2 and rat heart cells from IR injury (Hu et al., 2015; Zhou et al., 2018). Additionally, melatonin promotes OPA-1–mediated mitochondrial fusion by activating YAP, thereby reducing IR-induced mitochondrial apoptosis and cardiac IR injury (Ma and Dong, 2019). Several signaling pathways are involved in cardiac mechanotransduction, and molecules have been targeted to attenuate specific pathways to inhibit or reverse harmful mechanical stress–induced cardiomyopathy. Verteporfin inhibits the interaction between YAP and TEAD1 to deplete the downstream YAP signal. Therefore, verteporfin has therapeutic potential for pressure overload cardiac remodeling.

### Non-coding RNAs

In the last 10 years, ncRNAs have been studied worldwide, and several critical ncRNAs with essential biological functions have been identified. Antisense oligonucleotide therapy targeting strategies to modulate gene splicing have been used for ncRNA-based gene therapy. While lncRNA mimics have been used to express specific oligonucleotides (Lee J. et al., 2016), lncRNA-Plscr4 overexpression regulates mitochondrial function to attenuate hypertrophic stress by targeting miR-214 in mice-TAC or Ang-II–treated CMs (Lv et al., 2018; Zhang J. et al., 2019; Zhang M. et al., 2019). Additionally, silencing of lncRNA-Uc.323 or overexpression of lncRNA-Ahit could attenuate phenylephrine-induced CM enlargement through the mitochondrial pathway (Liu et al., 2016; Viereck et al., 2016; Yan et al., 2019; Sun et al., 2020).

### Gene Editing for Genetic Disorder

CRISPR/Cas9 has rapidly become one of the most popular and important approaches for genome editing because of its simplicity and adaptability. CRISPR/Cas9 and iPSC technology were recently applied to characterize Barth syndrome with mitochondrial abnormalities. The administration of the mitoTEMPO antioxidant to patient-derived iPSC-CMs efficiently regulated mitochondrial ROS production and sarcomere organization to rescue the pathological phenotype of Barth syndrome (Wang et al., 2014). Additionally, AAV technology has been developed to deliver genes to reverse cardiac function and restore mitochondrial function (Wang L. et al., 2020; Wang et al., 2021). AAV and lentivirus are the main viral tools for gene delivery for genetic editing. Diabetic mice induced by intraperitoneal streptozocin injections were randomized for treatment with lentivirus carrying Lin28a siRNA or Lin28a cDNA to knock down or overexpress Lin28a, respectively. Lin28a overexpression significantly decreased RhoA/ROCK signaling to alleviate mitochondria cristae destruction and promote heart function in diabetic mice (Sun et al., 2016). Additionally, Dkk1 adenovirus is transduced by intramyocardial injection, and Dkk1 overexpression aggravates Dox-induced CM apoptosis via the mitochondrial damage pathway (Liang et al., 2019). AAV serotype 9 (AAV-9) Plscr4, lncRNA-Plscr4 overexpression attenuates the hypertrophic response in hyperpressure-loaded CMs (Liu et al., 2020). Non-viral delivery approaches involve liposome- or nanoparticle-mediated oligonucleotide delivery to heart tissue (Ishikawa et al., 2013). Chen et al. showed that modified aYAP mRNA could induce CM regeneration in an IR mouse model, which demonstrated a promising way to induce transit gene expression to achieve gene therapy.

## Perspectives

In summary, biophysical and biochemical sensing pathways establish the fundamental mechanotransduction pathways that impact the regulation of mitochondrial homeostasis. Shear stress, cyclic stretch, and static strain are three major mechanical forces CMs are subjected to. Exceeding mechanical stress impairs mitochondrial function through regulating MQC, ATP production, ROS accumulation, and Ca^2+^ handling, leading to CM hypertrophy and dysfunction. Therefore, restoring mitochondrial function by shutting down the exceeding mechanotransduction pathway is a potential therapeutic strategy for cardiovascular diseases. In this review, we highlighted the biological processes of mechanotransduction pathways in regulating mitochondrial function in response to mechanical stress. We also provided a brief description of gene therapy delivery modes used to deliver treatment based on mechanical stress–induced mitochondrial dysfunction, providing new integrative insights into cardiovascular diseases. According to current evidence provided by this review on mechanotransduction in mitochondrial hemostasis, the future cut edge should be located in the following several perspectives. The first thing is to get better understanding of how biophysical mechanical stresses regulate mitochondrial function, as present studies revealed several molecular signals downstream of integrin-FAK–myosin–laminA/C pathway involved in nuclear mitochondrial genes expression. However, mitochondria are found to bind with F-actin, so that the changes of mitochondria are unclear when faced with the physical stress directly. Second, the biochemical sensor–related signaling is really limited to describe, especially the crosstalk between physical and chemical changes under exceeding mechanical stress. At last, to attenuate the abnormal mechanical stress to restore normal mitochondrial function aiming to reverse CMs' phenotype is a promising way. However, the risks of high dosage of AAV administration need to be noticed, and modified strategy is urgently required to be induced to reduce the supplement of AAV and achieve the same therapeutic goal at the same time. In generally, there is still a long way to obtain detailed understanding of mechanotransduction pathway in regulating mitochondrial function and to make targeting treatments.

## Author Contributions

HL and PY were primarily responsible for the Biological function of mechanical stress in cardiomyocytes, Molecules involved in mechanotransduction in CMs, and The common understanding of mitochondrial homeostasis sections, respectively. YQ and YY were primarily responsible for the Mechanical cues contribute to the maintenance of mitochondrial homeostasis, and Targeting mechanotransduction pathways to restore mitochondrial function sections, respectively. DZ and YL contributed equally to organize and edit the article. All authors contributed to the article and approved the submitted version.

## Conflict of Interest

The authors declare that the research was conducted in the absence of any commercial or financial relationships that could be construed as a potential conflict of interest.
